# Shaping meiotic chromosomes with SUMO: a feedback loop controls the assembly of the synaptonemal complex in budding yeast

**DOI:** 10.15698/mic2016.03.486

**Published:** 2016-02-19

**Authors:** Hideo Tsubouchi, Bilge Argunhan, Tomomi Tsubouchi

**Affiliations:** 1 Genome Damage and Stability Centre, Life Sciences, University of Sussex, Brighton BN19RQ, UK.; 2 National Institute for Basic Biology, National Institutes of Natural Sciences, Okazaki, Aichi 444-8585, Japan.

**Keywords:** budding yeast, chromosome, homologous recombination, meiosis, SUMO, SUMOylation, the synaptonemal complex

## Abstract

The synaptonemal complex (SC) is a meiosis-specific chromosomal structure in which homologous chromosomes are intimately linked through arrays of specialized proteins called transverse filaments (TF). Widely conserved in eukaryote meiosis, the SC forms during prophase I and is essential for accurate segregation of homologous chromosomes at meiosis I. However, the basic mechanism overlooking formation and regulation of the SC has been poorly understood. By using the budding yeast *Saccharomyces cerevisiae*, we recently showed that SC formation is controlled through the attachment of multiple molecules of small ubiquitin-like modifier (SUMO) to a regulator of TF assembly. Intriguingly, this SUMOylation is activated by TF, implicating the involvement of a positive feedback loop in the control of SC assembly. We discuss the implication of this finding and possible involvement of a similar mechanism in regulating other processes.

As a specialized cell cycle that produces gametes, meiosis is central to the continuity of life in sexually reproducing organisms. During meiosis, one round of DNA replication is followed by two rounds of nuclear division. Meiosis I is unique in that homologous chromosomes (homologs) are separated, unlike meiosis II or mitosis where sister chromatids are separated. For homologs to accurately segregate to opposite poles of the cells, they need to be physically attached to each other to ensure their biorientation at metaphase I.

In the process of establishing physical connections between homologs, meiotic chromosomes undergo a number of complicated processes during the lengthy prophase I. During prophase I, meiotic chromosomes develop a prominent structure called the SC which serves as a platform for efficient formation of crossovers between homologs. The SC consists of two lateral elements parallel to each other, each of which comprises a chromosomal core (axis) of one of the aligned homologs. The space between two lateral elements is occupied by arrays of TFs assembled perpendicularly to the lateral elements. Thus, understanding the mechanism of TF assembly is crucial for understanding SC formation.

Our previous work identified the Ecm11-Gmc2 complex as an activator for assembling Zip1, the yeast TF protein. Ecm11 becomes robustly conjugated to multiple SUMO molecules in prophase I, raising the possibility that Zip1 assembly is controlled by the levels of Ecm11 SUMOylation. Indeed we found a close correlation between the extent of Ecm11 SUMOylation and Zip1 assembly. In mutants where different SUMO-targeting sites of Ecm11 were mutated, chromosomal loading of Zip1 was compromised according to the level of reduction in SUMOylation. Similarly, in the absence of two SUMO E3 ligases, Siz1 and Siz2, a reduction in Ecm11 SUMOylation caused poor Zip1 assembly. On the contrary, under conditions where Ecm11 becomes hyper-SUMOylated (see below), Zip1 became highly aggregative, often forming one large assembly body unassociated with chromosomes called a polycomplex. These observations suggested that the level of Ecm11 SUMOylation dictates the “stickiness” of Zip1, leading to the proposal of a novel mechanism controlling SC formation.

Intriguingly, efficient SUMOylation of Ecm11 requires Zip1; Ecm11 SUMOylation was markedly reduced in the absence of Zip1. Further characterization revealed that the N-terminus of Zip1 was necessary for promoting robust Ecm11 SUMOylation. By producing meiosis-specific proteins in vegetative cells, the N-terminal third of Zip1 along with Ecm11 and Gmc2 were shown to be sufficient for supporting robust SUMOylation of Ecm11 and Zip1 assembly.

Taken together, Ecm11 SUMOylation promotes Zip1 assembly whereas Zip1 itself is necessary for promoting Ecm11 SUMOylation. This conclusion strongly argues that SC formation is controlled by a positive-feedback mechanism between Ecm11 SUMOylation and Zip1 assembly.

One of the substructures of the SC that has long been recognized using electron microscopy is the central element which runs in parallel to and equidistantly from two lateral elements in the central region of the SC. It was recently found that the N-terminus of Zip1, SUMO and Ecm11 all localize to the central element. Thus, we propose that the central element serves as a control center for regulating TF assembly.

SC formation initiates at discrete spots marked by the synapsis initiation complex (SIC) proteins. SIC formation occurs even in the absence of Zip1 although chromosomes remain unsynapsed. Interestingly, even under this condition, Ecm11/Gmc2 are recruited to the synapsis initiation sites. Once SC formation has started, Ecm11/Gmc2 are loaded to the central region of the SC like Zip1 while SIC proteins remain as punctate foci. Taken these localization patterns of Ecm11/Gmc2 into account, we propose that SC formation takes the following steps (Figure 1). First, both central element proteins (Ecm11/Gmc2) and Zip1 are recruited to the SIC independently, which allows the initial interaction between these proteins. This is followed by the nucleation of Zip1 and consequent activation of Ecm11 SUMOylation, which in turn promotes Zip1 assembly. These steps establish the positive feedback loop, leading to further loading/assembly of Zip1 and further recruitment of Ecm11/Gmc2 and Ecm11 SUMOylation.

**Figure 1:**
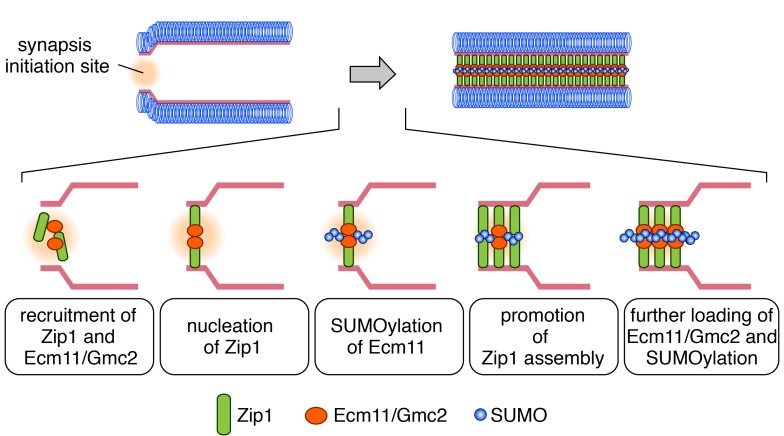
FIGURE 1: Mutual activation between Ecm11 SUMOylation and transverse filament assembly shapes the synaptonemal complex. We propose the following model regarding the mechanism forming the synaptonemal complex (SC). First, the transverse filament protein (Zip1) and the assembly activation factor (Ecm11/Gmc2) are independently recruited to a synapsis initiation site. Recruited Zip1 starts to assemble in conjugation with Ecm11/Gmc2, which promotes SUMOylation of Ecm11. This ignites the positive feedback loop, leading to further assembly of Zip1, loading of Ecm11/Gmc2 and SUMOylation of Ecm11 until the SC is fully established.

Currently we know little about the mechanism for terminating TF assembly. Given that the feedback loop relies on polySUMOylation of Ecm11, the simplest way to exit the loop is to discourage Ecm11 polySUMOylation, possibly by inhibiting SUMO ligases and/or activating SUMO proteases. To this end, we identified two proteins that are required for maintaining Ecm11 in an intermediate state of SUMOylation (i.e., Ecm11 becomes hyper-SUMOylated in their absence). The first protein is Ulp2, a SUMO protease, and the second is Zip3, a component of the SIC. While it makes sense that Ecm11 becomes hyper-SUMOylated in the absence of a SUMO protease, a role of Zip3 in downregulating SUMOylation was unexpected, given that Zip3 has previously been reported as a SUMO E3 ligase. It is possible that Zip3 functions as a regulator for determining the level of Ecm11 SUMOylation through controlling SUMO proteases.

An interesting feature of this model is in that the protein (i.e., Zip1) that constitutes the main component of a structure (i.e., the SC) promotes SUMOylation-mediated activation of its regulator (i.e., Ecm11). This system could be easily adapted to other contexts with the following elements: a recruiter, a structural protein with a SUMOylation-activation function, an assembly regulator activated by SUMOylation and a terminator with a deSUMOylation activity. A recruiter allows a structural protein to encounter its assembly activator, which ignites the positive-feedback loop that involves mutual activation of component assembly and SUMOylation of the activator. The feedback loop continues, thus shaping a structure until the formed macromolecule hits a target where a condition to suppress the loop is met, possibly through deSUMOylation of the activator.

